# Caribbean plate tilted and actively dragged eastwards by low-viscosity asthenospheric flow

**DOI:** 10.1038/s41467-021-21723-1

**Published:** 2021-03-11

**Authors:** Yi-Wei Chen, Lorenzo Colli, Dale E. Bird, Jonny Wu, Hejun Zhu

**Affiliations:** 1 Department of Earth and Atmospheric Science, University of Houston, Houston, USA; 2 Bird Geophysical, Houston, USA; 3 Department of Geosciences, University of Texas at Dallas, Richardson, USA

**Keywords:** Geodynamics, Geophysics, Tectonics

## Abstract

The importance of a low-viscosity asthenosphere underlying mobile plates has been highlighted since the earliest days of the plate tectonics revolution. However, absolute asthenospheric viscosities are still poorly constrained, with estimates spanning up to 3 orders of magnitude. Here we follow a new approach using analytic solutions for Poiseuille-Couette channel flow to compute asthenospheric viscosities under the Caribbean. We estimate Caribbean dynamic topography and the associated pressure gradient, which, combined with flow velocities estimated from geologic markers and tomographic structure, yield our best-estimate asthenospheric viscosity of (3.0 ± 1.5)*10^18^ Pa s. This value is consistent with independent estimates for non-cratonic and oceanic regions, and challenges the hypothesis that higher-viscosity asthenosphere inferred from postglacial rebound is globally-representative. The active flow driven by Galapagos plume overpressure shown here contradicts the traditional view that the asthenosphere is only a passive lubricating layer for Earth’s tectonic plates.

## Abstract (web summary)

Here, the authors follow a new approach using analytic solutions for Poiseuille-Couette channel flow to compute asthenospheric viscosities under the Caribbean. Active asthenospheric flow observed under the Caribbean contradicts the traditional view that the asthenosphere is only a passive lubricating layer for Earth’s tectonic plates.

## Introduction

The concept of a weak asthenosphere sandwiched between mobile tectonic plates above and a mechanically stronger sub-asthenospheric mantle below is fundamental for understanding plate tectonics^1,2^ and mantle convection^3,4^. Traditional viscosity constraints based on glacial isostatic adjustment (GIA) in Canada and Fennoscandia^5,6^ suggest an average viscosity of about 10^21^ Pa s for the upper half of the mantle^6^, with a mild viscosity reduction in the asthenosphere. While the viscosity reduction can be traded off against layer thickness^2,5,7^, these relatively high viscosity values imply a predominance of Couette-style flow where the asthenosphere, acting as a passive lubricating layer between tectonic plates and the underlying mantle, is sheared by the plate motions above (i.e., top-down driven asthenospheric flow)^8^. On the other hand, evidence exists for sub-plate asthenospheric flow that is decoupled from plate motions^9–12^ and actively drags the tectonic plates above (i.e., bottom-up driven flow)^13–15^. In contrast to Couette flow, this mechanism instead suggests active, pressure-driven Poiseuille flow of the asthenosphere induced by whole mantle convection. Such a scenario requires a thin and very weak asthenosphere, with lower viscosities that are outside the bounds of classic GIA studies. Indeed, recent viscosity estimates from post-seismic deformation^7^ and GIA in non-cratonic continental^16^ and oceanic areas^17–19^ suggest 2–3 orders of magnitude weaker asthenosphere.

**Fig. 1:**
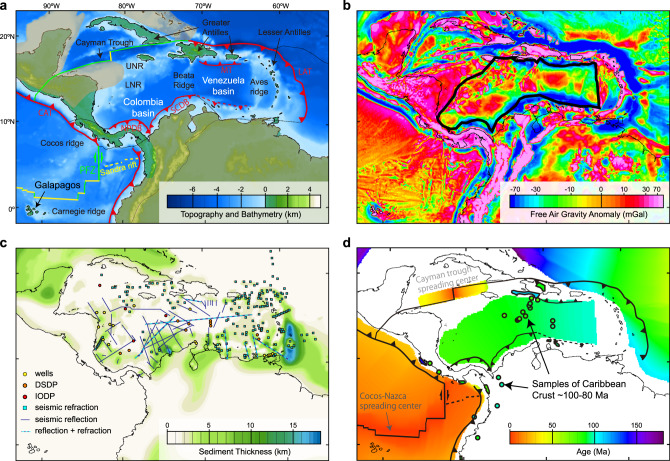
Maps of the Caribbean study area. **a** Topographic and bathymetry. Plate boundaries of Bird^73^ are colored-coded as active spreading centers (yellow lines); extinct spreading centers (yellow dashed lines); transform (green lines); subduction zones (red lines with teeth). LAT lesser Antilles trench, CAT central America trench, NPDB north Panama deformation belt, SCDB south Caribbean deformation belt, MT Muertos trench, PFZ Panama fracture zone, UNR upper Nicaraguan rise, LNR lower Nicaraguan rise. Dark orange shaded areas show continental crust^74^. **b** Satellite free air gravity anomaly^33^. The Caribbean oceanic plateau is bounded within the bold black line. **c** Sedimentary thickness constrained by seismic reflection, refraction, and borehole (see Supplementary Note 2 for references). **d** Age of the oceanic lithosphere from marine magnetic anomalies^75^ with Caribbean lithospheric ages slightly shifted to between 100 and 80 Ma based on recent dating results^30^^*,* and references therein^ (see “Methods” for details). Bold black lines are plate boundaries as in (**a**).

Eastward asthenospheric flow under the Caribbean (Fig. 1a) from the Pacific through the Panama slab window^20,21^ towards the Atlantic has been a long-standing hypothesis^22^ with geophysical^23–25^ and geological support^20,26^. Moreover, the Caribbean plate has been relatively fixed in a mantle reference frame since the Eocene^27^ and its current plate motion (V) is very low, <3 cm year^−1^ toward the west relative to a mantle reference frame^28^. This means that any eastward asthenospheric flow under the Caribbean plate if such exists, is unlikely to be passively driven by tectonic plate motions since the Caribbean plate is not fast-moving. Instead, any significant flow beneath the Caribbean would be mainly pressure-driven (i.e., Poiseuille flow) from the subsurface (i.e., bottom-up). Thus, the Caribbean region provides a unique opportunity to independently constrain the driving pressure, asthenospheric thickness, and flow velocity in one locality for the first time, allowing us to discriminate between Couette and Poiseuille flow and to obtain a significantly improved estimate of asthenospheric viscosity.

Here, we show our best-estimate asthenospheric viscosity of (3.0 ± 1.5)*10^18^ Pa s with the asthenospheric thickness of 200 ± 50 km, suggesting active, pressure-driven Poiseuille flow under the Caribbean. The asthenospheric viscosity obtained in this study should be roughly representative of large portions underneath oceanic and non-cratonic continental lithosphere, thus, challenging the traditional view that the asthenosphere is only a passive lubricating layer for tectonic plates.

## Results

In this study, we estimate the absolute viscosity of the asthenosphere from the pressure gradient and the asthenospheric flow velocities under the Caribbean. We use a simple analytical solution for planar Poiseuille-Couette flow^29^

1 $$u\left( y \right) = \frac{1}{{2\eta }}\frac{{\mathrm{d}}P}{{\mathrm{d}}x}y\left( {y - H} \right) + V\left( {{\mathrm{1 - }}\frac{y}{H}} \right)$$

where *u*(*y*) is the flow velocity as a function of depth, *η* is the asthenospheric viscosity, d*P* d*x*^−1^ is the pressure gradient, *V* is the upper plate velocity, and *H* is the channel thickness. The *x*-axis is positive to the east and the *y*-axis is positive downward. Higher pressure in the west thus yields a negative pressure gradient. Funneled by subduction zones and continental lithospheric roots (Fig. 1a), we argue the Caribbean region provides an ideal tectonic setting for measuring asthenospheric viscosity through a plane channel that closely approximates the conditions of the above analytical solution (Supplementary Note 1).

### Caribbean dynamic topography and pressure gradient

Deviations from hydrostatic pressure associated with mantle flow warp the surface of the Earth, adding a dynamic component of topography that we use to deduce a pressure gradient. The driving pressure gradient (d*P* d*x*^−1^) can thus be obtained by computing an isostatically-compensated residual basement depth, accounting for thermal subsidence of the lithosphere, sediment thickness, and crustal thickness (see “Methods” for details). We adopted a thermal age for the Caribbean lithosphere between 100 and 80 Ma (Fig. 1d) based on volcanic samples^30^ and references therein. In addition, we built an improved Caribbean sediment thickness map (Fig. 1c) by augmenting a global dataset with regional seismic reflection, refraction, and borehole data (see Supplementary Note 2 for details).

**Fig. 2:**
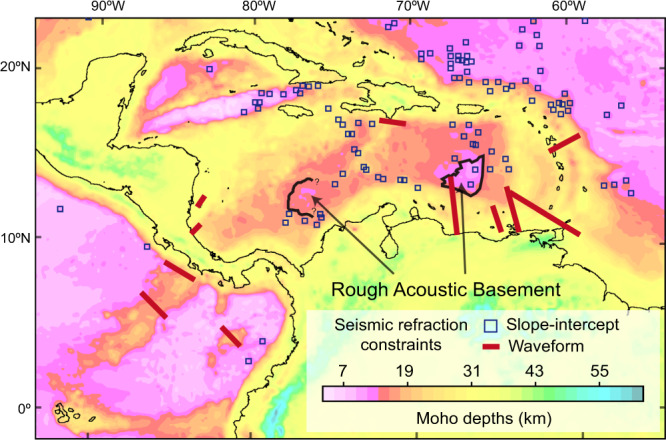
Moho depth map from this study based on the inversion of gravity and seismic constraints (shown by small squares). The two thicker black lines show the boundaries of the smooth- rough acoustic basement that reflect the transitions from over thickened to regular thickness oceanic lithosphere. For comparisons to other published Moho models, see Supplementary Fig. 2.

Caribbean crustal thicknesses are challenging to estimate because Caribbean crust is largely composed of an over thickened oceanic plateau^31^, the so-called Caribbean large igneous province (CLIPs) that erupted at the Galapagos hotspot during late Cretaceous times (Fig. 1d)^30,32^. This over thickened crust has hampered imaging of the base of the crust (i.e., Moho) via seismic reflection and refraction methods, resulting in limited crustal thickness constraints (Fig. 2 blue boxes). Therefore, we performed a structural inversion of free-air gravity anomalies (Fig. 1b) from the most recent version of satellite gravity data^33^. The seismic refraction constraints (Fig. 2) were then integrated to establish an improved Moho surface (Supplementary Note 3) that provides necessary details, in contrast to existing global models (Supplementary Note 4; Supplementary Fig. 2). Within the Caribbean (bounded by black lines in Fig. 1b), our model shows generally deeper Moho in the west and shallower Moho toward the east. Three known features can be independently identified: the Beata ridge, with shallower bathymetry (Fig. 1a) and deeper Moho (~22 km) (Fig. 2); the Colombia basin and the Venezuela basin, both with shallower Moho (~12 km) (Fig. 2). At Colombia and the Venezuela basins, seismic reflection studies^34,35^ identified two distinct ocean floors seismic characteristics: rough and smooth acoustic basements; these are interpreted as regular and plume-covered ocean floors, respectively. The boundaries between the two previously interpreted basement types (black lines in Fig. 2) imply a change of crustal thickness, which is highly consistent with our model (Fig. 2).

**Fig. 3:**
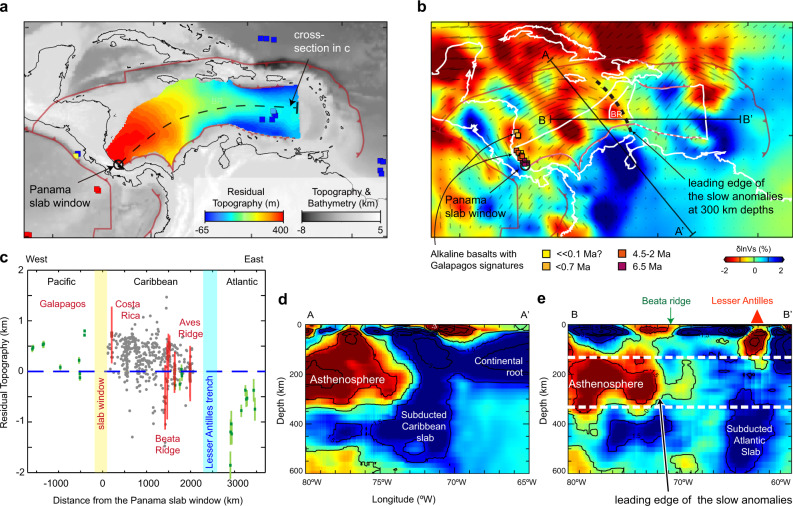
Caribbean dynamic topography and asthenospheric viscosity analyses. **a** Caribbean dynamic topography map from this study overlain on topography. Solid squares show residual topography from Hoggard, White^36^, which are color-coded to the same dynamic topography scale. Major plate boundaries are shown as red lines. A black open circle shows the location of the proposed slab window^21^. **b** Full-waveform tomography of Zhu et al.^25^ at 300 km depth. Gray lines show azimuthal anisotropy that may indicate mantle flow directions. We interpret the eastern edge of the slow anomalies near the Beata ridge (BR) as the leading edge of the hot mantle material passing through the Panama slab window. Colored squares show northward younging back-arc alkaline basalts with Galapagos isotopic signatures that have been interpreted as Galapagos-sourced asthenosphere passing through the slab window^26^. The black dashed line shows the present leading edge of the slow anomalies; the distance from the black dashed line to the Panama slab window is later used to obtain the flow velocities in Fig. 4. **c** Dynamic topography plotted as a function of the distance from the slab window shown in (**a**). Gray dots are obtained from this study (i.e., reflection-constrained basement and gravity-and-seismic constrained Moho). Red dots are robust estimations with both the basement and Moho constrained by seismic. The dynamic topography shows an eastward decreasing trend characterized by ~500 m uplift at the Galapagos, ~300 m uplift near the Panama slab window to ~0 m near the Aves ridge to the east, and 500 m subsidence within the Atlantic. This trend fits with the trend of dynamic topography outside the study area, shown by green dots from Hoggard, White^36^. **e**, **f** are two cross-sections that clearly show ~200 ± 50 km thick asthenosphere from the slower areas colored in red^25^. Profile locations are shown in (**b**). Panels (**b**), (**d**), and (**e**) are modified from Figs. 5 and 6 in reference ^25^.

Isostatically-compensated residual basement depths (Fig. 3a; Supplementary Fig. 4) show a clear cross-basin gradient (Fig. 3c) that is tilted downwards to the east. The western Caribbean shows considerable dynamic support (i.e., lies above the blue dashed line in Fig. 3c) whereas the eastern Caribbean shows no dynamic uplift or subsidence (Fig. 3c). A linear regression of all 9684 basement depth values within the Caribbean against distances from the Panama slab window yields a robust estimate for the large-scale dynamic topography gradient *A* = −0.14(1) m km^−1^ with a correlation coefficient *r*^2^ = −0.33 (see “Methods” for further details and uncertainty estimates). Our results are consistent with recent high-precision dynamic topography spot measurements^36^ and with reports of the Caribbean being in near-isostatic equilibrium near the Aves Ridge^37^, and improve a recent estimate of dynamic topography in the Caribbean region^38^, which was limited by an inadequate characterization of the local crustal structure.

The driving pressure gradient can be calculated from the gradient of the lithostatic pressure associated with the dynamic topography d*P* d*x*^−1^ = ∆*ρgA*, where ∆*ρ* is the density contrast between the mantle lithosphere and seawater (2270 kg m^−3^), and *g* is the acceleration of gravity (9.8 m s^−2^). Finally, we estimate the asthenospheric thickness (*H*) to be 200 ± 50 km based on a recent regional full-waveform tomographic model^25^ (Fig. 3d, e), consistent with similar observations made in the Pacific and Atlantic Oceans^39,40^. Because the asthenospheric thickness is independently constrained, the uncertainty originating from the tradeoffs between viscosity contrast and asthenospheric thickness^2,5,7^ is no longer a prominent concern.

### Pressure-driven asthenospheric flow underneath the Caribbean

**Fig. 4:**
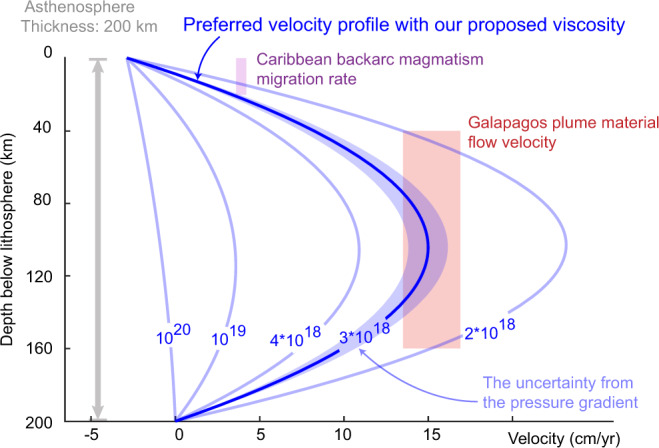
Profiles of Caribbean asthenospheric flow velocities versus depth as a function of asthenospheric viscosity (see Eq. (1)). The thick blue line denotes the profile corresponding to our preferred viscosity value of 3*10^18^ Pa s. The blue-shaded area shows the uncertainty of the velocity profile induced by the uncertainty in the pressure gradient. Colored boxes show independent estimates of flow velocity, constrained by the depth of melting and the migration rate of back-arc magmatism^26^ (purple box) and by the current position of the leading edge of the Galapagos plume material which has been flowing into the Caribbean since the opening of the slab window at ~8.5 Ma (red box). Profiles for alternative thickness values are reported in Supplementary Fig. 5.

Having estimated all other model parameters, Eq. (1) allows us to draw a family of flow velocity profiles as a function of asthenospheric viscosity (Fig. 4). For larger viscosity values (for example, 10^20^ Pa s in Fig. 4), Couette flow dominates and the asthenosphere is passively sheared to the west by Caribbean plate motions. For lower viscosity values, instead, Poiseuille flow dominates and the pressure gradient forces the asthenosphere eastwards. Our interpreted west-to-east directed flow is consistent with regional S-wave splitting measurements^23,24^ and azimuthal anisotropy^25^ (Fig. 3b). Age-progressive back-arc magmatism with clear Galapagos hotspot signatures in Central America provides further evidence of eastward mantle flows from the Pacific into the Caribbean region^20,26^ (Fig. 3b).

As a test, we use our estimated Western Caribbean dynamic uplift to predict the first-order temperature of the Galapagos-sourced mantle asthenosphere^41^:

2 $${{U = }}\frac{{{{H}}\alpha \left( {{{T - }}T_0} \right)}}{{{{1 - }}\alpha T_0}}$$

where *U* is the regional uplift due to dynamic topography (300 m), *H* is the thickness of the asthenosphere (200 km), *α* is the thermal expansion coefficient (3.3 × 10^−5^ °C^−1^), *T* is the average temperature of the Galapagos-derived material, and *T*_0_ is the ambient mantle temperature^42^ (1350 °C). We obtained *T* = 1393 °C, which is consistent with the mantle potential temperature of 1380–1450 °C obtained from the MgO content in the back-arc magmatism^26^. Our estimated average temperature, as well as the potential temperatures of the back-arc magmatism, are lower than the potential temperature of the Galapagos hot spot (1400–1500 °C)^42^, which is expected given the ~1500 km distance between the Galapagos and the slab window. It is also worth noting that our estimate is only ~50 °C warmer than ambient mantle^42^. The influx of this warmer-than-ambient mantle material in the asthenosphere is imaged by seismic tomography as a slow shear wave velocity anomaly (Fig. 3d, e) underneath the western Caribbean (Fig. 3b).

**Fig. 5:**
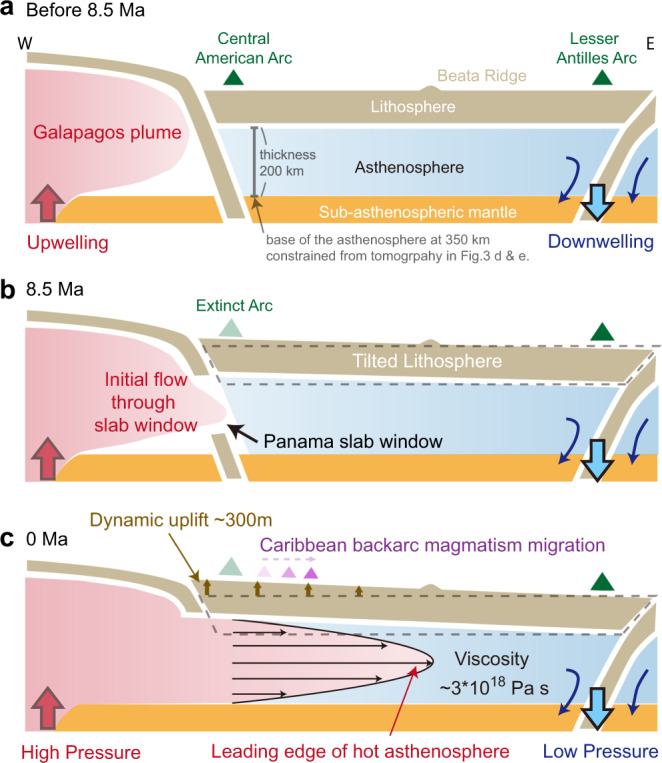
Time-evolution cartoon of Caribbean asthenosphere and dynamic topography. Hotter asthenosphere sourced from the Galapagos plume flowed eastwards through the Central America slab window towards the Caribbean and produced an eastward dynamic tilt of the Earth’s surface. **a** At 8.5 Ma, prior to the slab window opening, the regional upper mantle was segmented by subducted slabs. **b** At ~8.5 Ma, the opening of the Panama slab window allowed eastward asthenospheric flows that were driven by pressure differences between the upwelling Galapagos plume to the west, and the subduction downwelling to the east. This geological event terminated the arc at the Panama land bridge and began the dynamic uplift of the Caribbean. **c** At present, the leading edge of hot asthenosphere imaged by full-waveform tomography^25^ has reached the Beata ridge. Eastward propagation of hot asthenosphere across the region produced time-transgressive back-arc magmatism within Central America that has a clear Galapagos isotopic signature^26^. Integration of these space-time constraints allows us to obtain the flow velocity of the Galapagos hot material which, together with the independent estimates of the driving pressure gradient and the thickness of the asthenosphere, constrain the absolute viscosity of the asthenosphere under the Caribbean to ~3*10^18^ Pa s.

Our results show more details than previously known about the present-day vertical (dynamic topography) and horizontal (pressure-driven flow) manifestations of mantle pressure gradients under the Caribbean. The onset of the mantle pressure gradient can probably be traced to ~8.5 Ma when the Panama slab window formed^20,21^, and opened the mantle gateway between the Pacific and Caribbean (Fig. 5). The earliest backarc magmatism with Galapagos isotopic signatures began at 6.5 Ma in Costa Rica, and shows an age-progression northwards at a rate of 4 cm year^−1^, reaching Nicaragua at the Present-day^20,26^. In addition, there is evidence that Central America was uplifted ~500 m during late Miocene times, and this has been linked to the blockage of the Central America Seaway and the strengthening of the Atlantic Meridional Overturning Circulation at 9 Ma^43^. Significant non-tectonic uplift of the mountains in northern Colombia and Venezuela since late Miocene times have also been reported^44^. Although the mechanism for this uplift has not been established, it is similar in magnitude to our estimate for the present-day residual basement depth, suggesting these events had a major bottoms-up contribution from the mantle (i.e., dynamic topography). Similar dynamic uplift has also been proposed to influence ocean circulation in the North Atlantic^45^.

It is important to notice the uncertainties in velocity profile stemming from the dynamic topography gradient (blue shaded area in Fig. 4) and the asthenosphere thickness (Supplementary Fig. 5) are relatively minor. This allows us to use an estimate of asthenospheric flow velocity to place rather tighter constraints on the viscosity than previous studies, which have allowed its current orders-of-magnitude uncertainty.

### Independent constraints on the flow velocity and our preferred asthenospheric viscosity

A first estimate comes from the rate of propagation of the back-arc magmatism, together with its depth of generation^26^, which constrain the asthenospheric flow velocity at ~20 km below the lithosphere-asthenosphere boundary to be 4 cm year^−1^ (magenta box in Fig. 4). We argue for a limited role of slab rollback on the back-arc magmatism propagation, given that the arc has been relatively stationary since the late Miocene^46^ and that overriding central America is relatively fixed within a mantle reference^27^. A second constraint can be placed by considering that the slow seismic anomaly underneath the Caribbean (Fig. 3b) only extends to the Beata ridge (Fig. 3b, e). We then interpreted the edge of the slow seismic velocity anomaly as the leading edge of the Galapagos hot material that flowed through the Panama slab window from the Pacific since ~8.5 Ma. Assuming a steady flow, we obtained an average peak flow velocity of ~15 cm year^−1^ within the bulk of the asthenosphere (red box in Fig. 4). Flow velocities of similar magnitude have been reported for the North Atlantic^41^.

These two estimates constrain the asthenospheric viscosity under the Caribbean to (3.0 ± 1.5)*10^18^ Pa s (Fig. 4). This value is significantly lower (i.e., weaker) than post-glacial rebound estimates (10^20^ Pa s) for cratonic regions^5^, but is consistent with independent estimates for non-cratonic and oceanic regions^7,16–19^.

## Discussion

### Global applicability of estimated Caribbean viscosity

The asthenosphere under the Caribbean is plume-fed (Figs. 3 and 5) and slightly warmer (~50 °C) than the ambient mantle (Eq. (2) and “Methods”). How much of Earth’s asthenosphere is plume fed remains an open question^47^, but our results show that the plume material under the Caribbean plate is far-traveled, the Beata ridge being ~3000 km away from the Galapagos. Moreover, the average excess temperature of the asthenosphere underneath the western portion of the Caribbean plate can only reduce viscosities by a factor of ~3 (see “Methods”). This suggests that our Caribbean asthenospheric viscosity estimate is not overly affected by the slightly elevated temperatures (~50 °C above ambient), and should be roughly representative (within a factor of ~3) of large portions underneath oceanic and non-cratonic continental lithosphere, which is about ~60% of Earth. This further challenges the hypothesis that classic post-glacial rebound estimates are widely-applicable outside of cratonic regions.

### Implications for global Poiseuille flow in the asthenosphere

Our results have profound implications on mantle dynamics and plate tectonics. The thin and low-viscosity asthenosphere shown here indicates a pressure-driven channel flow^1^ that explains the long-wavelength pattern of mantle flow observed on Earth^3,4^. Dynamic topography gradients of comparable magnitude to our Caribbean results are reported in all oceanic basins^36^; thus, in many places asthenospheric flow speeds should be several cm year^−1^ ^48^, faster than plate velocities, challenging the paradigm of plate-driven asthenospheric flow (top-down). Instead, the concentration of horizontal asthenospheric flows leads to increased basal shear at the lithosphere-asthenosphere boundary^1^, despite the reduction in viscosity. Importantly, the basal shear is not just a passive drag due to Couette flow, as usually assumed^8^, but rather an active component of the tectonic torque balance due to the magnitude and variability of the Poiseuille component (see also “Methods”). Indeed, our results corroborate recent evidence showing that the waxing and waning of dynamic topography through time correlates with rapid changes in plate motions, as both are caused by variations in the strength of pressure-driven asthenospheric flow^14,15^.

## Methods

### Dynamic topography deconvolution

Dynamic topography reflects the topography due to transient viscous stresses caused by mantle upwellings or downwellings. In order to obtain the dynamic topography, we need to correct the total topography and bathymetry for the effects of lateral variations in the thermal age of the lithosphere, the crustal structure, and flexural effects. The remaining topography, also known as residual topography, would reflect the convective stresses caused by mantle convection.

We used our gravity-and-seismic constrained crustal and lithospheric structure (Supplementary Note 2, Note 3, and Supplementary Table 2). The isostatic correction for sediments^49^ (Sc) is calculated using

3 $${\mathrm{Sc = }}\frac{{\rho _m - \rho _s}}{{\rho _m - \rho _w}} \,*\, {\mathrm{ST}}$$

where *ρ*_*m*_, *ρ*_*s*_, and *ρ*_*w*_ are the densities of the mantle (3.3 g cc^−1^), the sediments (1.5–2.7 g cc^−1^, see the previous section for details), and seawater (1.03 g cc^−1^), respectively and ST is the total sediment thickness.

The isostatic correction for the crust^50^ (Cc) is calculated using

4 $${\mathrm{Cc = }}\frac{{\rho _m - \rho _c}}{{\rho _m - \rho _w}} \,*\, \left( {{\mathrm{CT}} - {\mathrm{C}}\mathrm{T}_0} \right)$$

where *ρ*_*c*_ is the density of the crust (2.85 g cc^−1^), CT is the crustal thickness at each grid point and CT_0_ is the average crustal thickness of the oceanic crust (7.1 km)^51^.

The residual topography (RT) is finally given by Eq. (5), for which depths are positive downwards:

5 $$\mathrm{RT} = d_{age}--\left( {d + \mathrm{Sc} + \mathrm{Cc}} \right)$$

where dage is the water-loaded basement depth expected from the lithospheric age based on the cooling model^52^ and d is the observed bathymetry. The residual topography (RT) obtained in Eq. (5) is the combination of flexural isostasy and dynamic topography. We then applied a second-order Butterworth low-pass filter with 1200 km corner wavelength to remove most of the contribution of flexural isostasy^53^ (Fig. 2b. See Supplementary Fig. 4 for alternative filtering choices).

A recent estimate of dynamic topography in the Caribbean region^38^ found a strong regional minimum in the middle of the plate, which is at odds with our results regardless of the filtering strategy used (Supplementary Fig. 4). Their work used the crustal model Crust1.0^54^, which is not sufficiently accurate within the Caribbean even for wavelengths >1000 km (Supplementary Fig. 2) because of the uneven and sparse distribution of the refraction data it is built upon. Biases in crustal thickness propagate into the isostatic correction, resulting in misinterpretations of dynamic signal, as noted previously^55^.

### Uncertainty of dynamic topography

The uncertainty in the dynamic topography propagates from each element in Eq. (5). Although each element has its own uncertainty, the main uncertainty comes from four sources—sediment thickness (ST), sediment density (*ρ*_*s*_), crustal thickness (CT), and crustal density (*ρ*_*c*_) (Supplementary Table 3), with the rest of the elements considered as constants because their uncertainties are relatively small. We estimate the uncertainty in dynamic topography at each gridpoint by propagating uncertainties (variances) and covariances as follows:

6 $$\left( {\sigma _{{\mathrm{RT}}}} \right)^2 =	 \left( {\frac{{\partial {\mathrm{RT}}}}{{\partial {\mathrm{ST}}}}} \right)^2\left( {\sigma _{{\mathrm{ST}}}} \right)^2 + \left( {\frac{{\partial {\mathrm{RT}}}}{{\partial \rho _s}}} \right)^2\left( {\sigma _{\rho _s}} \right)^2 + \left( {\frac{{\partial {\mathrm{RT}}}}{{\partial \rho _c}}} \right)^2\left( {\sigma _{\rho _c}} \right)^2 + \left( {\frac{{\partial {\mathrm{RT}}}}{{\partial {\mathrm{CT}}}}} \right)^2\left( {\sigma _{{\mathrm{CT}}}} \right)^2 \\ 	+ 2\left( {\frac{{\partial {\mathrm{RT}}}}{{\partial \rho _s}}} \right)\left( {\frac{{\partial {\mathrm{RT}}}}{{\partial {\mathrm{ST}}}}} \right)(\sigma _{\rho _s\,{\mathrm{ST}}}) + 2\left( {\frac{{\partial {\mathrm{RT}}}}{{\partial \rho _s}}} \right)\left( {\frac{{\partial {\mathrm{RT}}}}{{\partial {\mathrm{CT}}}}} \right)(\sigma _{\rho _s\,{\mathrm{CT}}}) + 2\left( {\frac{{\partial {\mathrm{RT}}}}{{\partial \rho _c}}} \right)\left( {\frac{{\partial {\mathrm{RT}}}}{{\partial {\mathrm{CT}}}}} \right)(\sigma _{\rho _c\,{\mathrm{CT}}}) \\ 	+ 2\left( {\frac{{\partial {\mathrm{RT}}}}{{\partial {\mathrm{ST}}}}} \right)\left( {\frac{{\partial {\mathrm{RT}}}}{{\partial {\mathrm{CT}}}}} \right)(\sigma _{{\mathrm{ST}}\,{\mathrm{CT}}}) + 2\left( {\frac{{\partial {\mathrm{RT}}}}{{\partial \rho _m}}} \right)\left( {\frac{{\partial {\mathrm{RT}}}}{{\partial {\mathrm{CT}}}}} \right)(\sigma _{\rho _m\,{\mathrm{CT}}})$$

The sediment thickness (ST) was obtained by multiplying the reflection-based travel time and the refraction-based velocity. The travel time measurements have negligible uncertainty, so the uncertainty of the sediment thickness comes mainly from the heterogeneity of the refraction-based velocity structure across the Caribbean (Supplementary Fig. 1a). Such heterogeneity might come from local interbedded basalt, limestone, or unconformities. By regressing all available measurements where seismic refraction and reflection are both conducted^56–60^, we obtained that the uncertainty in sediment thickness as a function of travel time is 0.4 km s^−1^ (Supplementary Fig. 1a, b). We then obtained the sediment thickness and the corresponding uncertainty at each grid point.

The sediment density (*ρ*_*s*_) was obtained from a density–depth function regressing from the index property density of IODP boreholes in the Caribbean^61^ (Supplementary Fig. 1c red curve; see Supplementary Note 3 for details). To estimate the uncertainty, we randomly generated 5,000 density profiles and analyzed their statistics. The upper 1.2 km of each density profile was generated by resampling the IODP database while below 1.2 km, where the index property density is unavailable, we assumed the density is normally distributed around the best-fitting curve using the same standard deviation as the IODP density index. For each density profile, we then computed average sediment densities at any given sediment thickness from 0.1 to 15 km. The average of the 5000 synthetic average densities at any given thickness (black dots in Supplementary Fig. 1e) is consistent with the analytical solution (Supplementary Fig. 1e blue curve), showing that our best-fitting curve is not biased at any depths. The standard deviation of the synthetic average densities is assigned to be the uncertainty of average sediment density at each grid point (Supplementary Fig. 1f). The uncertainty decreases as the thickness increases, because the thicker the sediment layer, the more sediments that reach the maximum density due to compaction, resulting in a more stable average density.

The uncertainty of the crustal density (*ρ*_*c*_) comes directly from the density heterogeneity of global seamounts and oceanic plateaus, which Tetreault and Buiter^62^ estimated as 2.85 ± 0.12 g cc^−1^.

The uncertainty of the crustal thickness is difficult to estimate, especially when seismic and gravity are jointly used to constrain the Moho. We thus performed two independent analyses, both of which yield similar estimates. We started by estimating the quality of our gravity-constrained Moho (i.e., without the aids of seismic constraints) by comparing it to the published refraction Moho, which includes two types of experiments: vintage experiments using the slope-intercept method and newer wide-angle experiments using waveform modeling. The average difference between published refraction Moho depths and our gravity-constrained Moho depth is −0.026 km with a standard deviation of 2.16 km.

As the uncertainty of the seismically estimated Moho depth is a function of crustal thickness, we plotted the two Moho estimations against each other (Supplementary Fig. 3) with given uncertainties. In the Caribbean, recent studies with waveform-modeled experiments^58,60,63–65^ suggest the uncertainty of the Moho is within ±2 km, about ~10% of the crustal thickness, which is consistent with the uncertainty estimation of refraction-constrained crustal thickness in global oceans^66^. The slope-intercept method^31,67,68^ typically underestimates crustal thickness by ~20%^51^. Therefore, we assigned 10% uncertainty to waveform-modeled Moho and 20% to slope-intercept-modeled Moho (Supplementary Fig. 3). The comparison shows our gravity constrained Moho is highly consistent with the published refraction Moho with a reduced ***χ***^2^ equal to 1.09. Our gravity-and-seismic constrained Moho in the Caribbean has uncertainty at most as high as the one obtained by seismic refraction studies, which is about ~10% of the crustal thickness, yielding an average uncertainty in the Moho depth of 1.3 km.

As an additional estimate of the uncertainty in the Moho depth, we performed a number of synthetic inversions where we modified one of the four input grids by adding a zero-mean Gaussian error with a standard deviation equal to the uncertainty obtained in the previous paragraphs. The results are summarized in Supplementary Table 3. As noted in previous works^69^, the uncertainty in crustal density (i.e., the density contrast across the Moho) has the largest effect on the Moho depth, while the uncertainties in sediment thickness and sediment density give a much smaller contribution. Moreover, the uncertainties in sediment thickness and sediment density have a strong covariance, which reduces their composite effect. This result is not surprising, because the thicker the sediment, the higher the uncertainty of its thickness, but the lower the uncertainty of its average density (see Supplementary Note 3 for details). The average uncertainty in Moho depth estimated via this synthetic analysis is 1.1 km, in line with our previous estimate.

Using now Eq. (6), we found the uncertainty in the dynamic topography at the Caribbean is 0.1–1.3 km with an average of 0.4 km. The main source of uncertainty comes from the uncertainty in the Moho depth. To obtain the uncertainty of the dynamic topography gradient, we regressed all 9684 point estimates of residual basement depth within the Caribbean against distance from the slab window, which yields a linear gradient of *A* = −0.14(1) ± 0.01 m km^−1^. difference of dynamic topography across the Caribbean within one standard deviation is ~300 ± 20 m.

### Uncertainty of asthenospheric flow velocity

The uncertainty of the flow velocity (*σ*_*U*_) comes from the uncertainty in the age of the opening of the slab window (*σ*_SW_) and the uncertainty in the location of the leading edge of the slow anomaly, which is due to the horizontal resolution of seismic tomography (*σ*_HR_):

7 $$\left( {\sigma _U} \right)^2 = \left( {\frac{{\partial {{HR}}}}{{\partial U}}} \right)^2\left( {\sigma _{{{HR}}}} \right)^2 + \left( {\frac{{\partial {{SW}}}}{{\partial U}}} \right)^2\left( {\sigma _{{{SW}}}} \right)^2$$

Since the highest frequency used in the tomography model, US32 is 15 s^25^, and the shear wave speeds are between 4 and 5 km s^−1^, the minimum wavelength is between 60 and 75 km. Although full-waveform inversion enables us to achieve the nominal (theoretical) resolution as half of the wavelength, the spatial resolution also depends on data coverage and data quality. The point spread function tests at the depth of 350 km^25^ reveal good recovery of Gaussian anomalies with a half-width of 120 km. We, therefore, conclude that 120 km would be a conservative estimate of the horizontal resolution *(σ*_*HR*_) in the central Caribbean. The age of the opening of the slab window is between 8 and 9 Ma, before the cessation of the arc magmatism in southern Costa Rica at 8 Ma^20^, and after the onset of the Panama fracture zone (PFZ in Fig. 1a) at 9 Ma (see Supplementary Note 1 for details). Therefore, we assigned a ±0.5 Ma uncertainty (*σ*_*sw*_) for the age of the opening of the slab window at 8.5 Ma. Collectively, we obtained the peak flow velocity to be 15.2 ± 1.7 cm year^−1^.

### Uncertainty of asthenospheric viscosity

The uncertainty of the asthenospheric viscosity (*σ*_*η*_) propagates from each element in Eq. (1). The current plate motion (*V*) of 2.875 cm year^−1^ ^28^ is assumed to have negligible uncertainty. The main uncertainty comes from the pressure gradient (${\sigma _{{{\mathrm{d}P}}\,{{\mathrm{d}x}}^{{{ - 1}}}}}$), the channel thickness (*σ*_*H*_), and flow velocity (*σ*_*U*_):

8 $$\left( {\sigma _\eta } \right)^2 = \left( {\frac{\partial }{{\partial \eta }}\left( {\frac{{\mathrm{d}}P}{{\mathrm{d}}x}} \right)} \right)^2\left( {\sigma _{{{\mathrm{d}}P}\,{{\mathrm{d}}x}^{ - 1}}} \right)^2 + \left( {\frac{{\partial H}}{{\partial \eta }}} \right)^2\left( {\sigma _H} \right)^2 + \left( {\frac{{\partial U}}{{\partial \eta }}} \right)^2\left( {\sigma _U} \right)^2$$

The uncertainty of the flow velocity (*σ*_*U*_) is 1.7 cm year^−1^ (see the previous section for details). The uncertainty of the pressure gradient (${\sigma _{{{\mathrm{d}P}}\,{{\mathrm{d}x}}^{{{ - 1}}}}}$) is ~10% of the pressure gradient, which stems from the uncertainty of the dynamic topography gradient (see the previous section for details). The uncertainty of the channel thickness (*σ*_*H*_) comes from the vertical resolution of the tomography, which is estimated to be ±50 km. Collectively, we obtained the asthenospheric viscosity to be (3.0 ± 1.5)*10^18^ Pa s.

### Basal shear induced by asthenospheric flow

The shear stress (*σ*) produced by the asthenospheric flow at the base of the lithosphere can be calculated directly from the velocity and viscosity of the asthenosphere flow:

9 $$\sigma = \frac{H}{2}\left( {\frac{{{\mathrm{d}}P}}{{{\mathrm{d}}x}}} \right){\mathrm{ + }}\eta \frac{V}{H}$$

With our best estimates of all the parameters, the basal shear is −0.33 ± 0.08 MPa (i.e., the asthenosphere is dragging the Caribbean plate eastward), in line with estimates based on calculations of tectonic force balance^70^.

### Volume flux through the slab window

An estimate for the volume flux of asthenospheric material through the slab window can be obtained by multiplying the average flow velocity by the cross-sectional area of the slab window. We compute an average flow velocity by integrating Eq. (1) and dividing the result by the thickness of the asthenosphere, which yields

10 $$V_{{\mathrm{avg}}} = \frac{{ - H^2}}{{{\mathrm{12}}\eta }}\left( {\frac{{{\mathrm{d}}P}}{{{\mathrm{d}}x}}} \right) + \frac{V}{2} = {\mathrm{\sim 9}}{\mathrm{.5}}\,{\mathrm{cm}}\,{\mathrm{yr}}^{{\mathrm{ - 1}}}$$

The cross-sectional area of the slab window^24^ between 150 and 350 km is ~6*10^10^ m^2^, giving a volume flux of ~185 m^3^ s^−1^.

### Viscosity reduction due to excess temperature

The temperature dependency of the viscosity of mantle rocks is usually parametrized with an Arrhenius-type law:

11 $$\eta \left( {p,T} \right){\mathrm{\sim exp}}\left( {\frac{{E + {\mathrm{pV}}}}{{{\mathrm{RT}}}}} \right)$$

where *p* is the pressure, *T* is the temperature, *R* is the gas constant, *E* is the activation energy and *V* is the activation volume. The increase in viscosity associated with a decrease in temperature from *T*_1_ = 1393 °C to *T*_2_ = 1350 °C can then be expressed as:

12 $$\frac{{\eta _2\left( {p,T_2} \right)}}{{\eta _1\left( {p,T_1} \right)}} = {\mathrm{exp}}\left[ {\left( {E + {\mathrm{pV}}} \right) \,*\, \frac{{(T_1 - T_2)}}{{RT_1T_2}}} \right]$$

Using recent estimates of 500 kJ mol^−1^ for *E*^71^, 15 cm^3^ mol^−1^ for *V*^72^ and a pressure of 8 GPa (corresponding to ~245 km depth) yields a ratio of 3.27.

## Supplementary information

Supplementary Information

## Data Availability

All data generated or analyzed during this study are included in this published article (and its supplementary information files). Source data are provided with this paper.

## Source data

Source Data

## Code availability

The Oasis Montaj code used to generate the crustal model is a commercial code that can be assessed through the code developers, Geosoft.
