# Supplementary material for: Caribbean plate tilted and actively dragged eastwards by low-viscosity asthenospheric flow
Source: Nat Commun. 2021 Mar 11;12:1603. doi: 10.1038/s41467-021-21723-1 (PMC7952903; doi:10.1038/s41467-021-21723-1)
Supplement: Supplementary file 1 — Supplementary Information [file 41467_2021_21723_MOESM1_ESM.pdf]

Supplementary Information for

"Caribbean plate tilted and actively dragged eastwards by low-viscosity asthenospheric flow"

Yi-Wei Chen[1,*], Lorenzo Colli[1], Dale E. Bird[1,2], Jonny Wu[1] and Hejun Zhu[3]

[1] Dept. of Earth and Atmospheric Science, University of Houston.

[2] Bird Geophysical.

[3] Dept. of Geosciences, University of Texas at Dallas.

*To whom correspondence should be addressed; E-mail: yiweichen.tw@gmail.com

Supplementary Note 1: A plane-channel-like Caribbean upper mantle

The Caribbean, being bounded by continental roots and subduction zones (Fig. 1 a), is reminiscent of a relatively simple plane channel. This special tectonic setting blocks most possible paths underneath the Caribbean, funneling the asthenosphere to flow generally to the east, and was established before the opening of the Panama slab window. In the following discussion, we will first summarize the initiation age of the circum-Caribbean subduction systems. Then we will review the activation age of the asthenospheric flow underneath the Caribbean due to the opening of the slab window at Panama.

To the east, the Caribbean is bounded by the Lesser Antilles subduction zone (LAT on Fig.1a), where the Atlantic is currently subducting underneath the CLIPs. This subduction system, forming since 40 Ma in response to a potential slab roll-back [1], originally belonged to a longer and older subduction system along the whole leading edge of the Caribbean, the Greater Antilles subduction system. Due to the eastward motion of the CLIPs and the continuous westward motion of the Americas, the proto-Caribbean, an oceanic basin formed during the separation of the Americas, was subducted underneath the CLIPs [2, 3], forming the Greater Antilles arc (Cuba, Hispaniola, Puerto Rico, Jamaica, and the Aves ridge) between 70 and 45 Ma on the eastern edge of the Caribbean .

At the southern edge, the CLIPs subducted underneath South America, forming the South Caribbean deformation belt (SCDB in Fig.1a). Subduction is believed to have initiated first in Colombia in the Paleocene, as the earliest arc-related granitoids was found at the continental margin and dated at 58 Ma [4], and then have progressively propagated eastward: from middle Eocene in western Venezuela to early Miocene in central Venezuela, and moving farther eastward during late Neogene time. Such west-to-east initiation of subduction was recorded in the deposition and distribution of sedimentary megasequences of the SCDB [5]. Besides the SCDB, there are two potential subduction systems directly related to the subduction of the CLIPs: to the southwest, the North Panama deformation belt (NPDB in Fig. 1a) and to the northeast, the Muertos trench (MT in Fig. 1a). However, due to the limited subduction of short slabs (~100 km), they play a less important role, funneling the asthenospheric flow.

To the west, the Caribbean is bounded by the Central America subduction zone (CAT in Fig. 1a), which was initiated slightly after the main phase of eruption of the CLIPs in late Cretaceous. When the CLIPs moved eastward with the Farallon plate [2, 3], they collided with the westward-moving Americas at 75-72 Ma [6, 7]. Slightly after the collision, the eastward moving Farallon plate formed a new subduction system at the trailing edge (western edge) of the Caribbean, which was recorded as the earliest juvenile arc in Panama at 71-69 Ma [8]. At ~27 Ma, the Farallon plate broke up into the Cocos and Nazca plates, separated by a new spreading ridge. Continuous arc magmatism in both Costa Rica (Cordillera Talamanca) [9] and in Panama (Cordillera Central) [8] suggests that there was no break in subduction under the western margin of the Caribbean plate.

At ~8.5 Ma, a reorganization of the Cocos-Nazca spreading ridge led to a regional plate reorganization. According to the magnetic anomalies [10] adjacent to the now-extinct Sandra Ridge (Fig. 1a), this previous spreading center was abandoned at 9 Ma, and the plate boundary shifted to the Panama fracture zone (PFZ in Fig. 1a). To the east of the PFZ, the lithosphere which had originally belonged to the Cocos plate was captured by the Nazca plate, subducting to the east, while the Cocos plate west of the PFZ kept subducting to the north. The plate boundary from Costa Rica to Panama thus shifted from a subduction zone to a transform margin [11]. Such plate boundary change is also recorded in the cessation of arc magmatism after 8 Ma at the Cordillera Talamanca in Costa Rica [9] and after 9-10 Ma at Cordillera Central of Panama [8]. This plate reorganization generated a slab window in the mantle [12], opening a gate for hot Pacific mantle materials to flow through, as evidenced by the appearance of backarc magmatism with clear Galapagos hotspot geochemical signature since 6.5 Ma [13] and by the adakites found in Panama, which are interpreted as melting of the slab edges around the slab window [8, 13].

Supplementary Note 2: Seismic-constrained basement depth

Basement depths can be obtained by subtracting a published global grid of sediment thickness [14, 15] from topography [16] and bathymetry [17]. However, the resolution of such global grids is insufficient and their uncertainty is too high for the purpose of computing residual basement depths. Here we improve the fidelity of such global grids using the wealth of seismic reflection [5, 18, 19, 20, 21, 22, 23, 24, 25, 26, 27, 28, 29, 30, 31, 32, 33], refraction [28, 29, 30, 31, 32, 33, 34, 35, 36, 37, 38, 39] and

borehole data [26, 40, 41, 42, 43, 44, 45] for the Caribbean that has recently been published in crustal-scale potential fields studies. We summarized the two-way travel time data from published seismic reflection in the Caribbean [5, 18, 19, 20, 21, 22, 23, 24, 25, 26, 27, 28, 29, 30, 31, 32, 33]. In those locations where both seismic refraction and reflection studies were conducted [31, 32, 33, 34, 46], we were able to obtain basement depths from reflection-based travel time data through the refraction-based velocity structure, allowing us to improve accuracy.

Supplementary Note 3: Gravity-and-seismic constrained Moho

We use the most recent version of satellite gravity data [47] that more accurately capture the details of the crustal structure in the Caribbean (Fig.1b). We assumed that the lateral free-air gravity anomaly variations can be fully explained by the lateral mass variations integrated from a simplified 3D model with four distinct gridded layers - sea water, sediments, crust and upper mantle with a vertical extent of 300 km. The mass of each layer is calculated by multiplying their thickness with density, which are either independently constrained or are defined by previous studies.

Topography and bathymetry are taken from GTOPO30 [16] and TerrainBase Global Terrain Model [17], respectively. We avoided the higher-resolution data of the ETOPO1 [48], because a gravity analysis was included in the model generation process. The density of the sea water is assigned as 1.03 g cc^{-1}. The uncertainty in topography and bathymetry are assumed to be negligible.

The sediment thickness at the Caribbean is taken from the differences between bathymetry and basement depths as described in the previous section. Beyond the Caribbean, published grids of global sediment thickness was used on both the continents [14] and the ocean [15]. The density of sediments increases with depths as they are buried and compacted. We parametrize the increase in density with depth as an exponential decrease in porosity:

$$\rho_s = \rho_{sg} + \phi_0(\rho_w - \rho_{sg}) * \exp(-Z/\lambda) \qquad (13)$$

where Z is the depth below the seafloor, ϕ_0 is the zero-depth porosity, λ is the compaction decay length, ρ_w=1.03 g cc^{-1} is the density of sea water and ρ_{sg} is the grain density. Most sediments in the Caribbean are either quartz (grain density = 2.65 g cc^{-1}) or more commonly calcite [42] (grain

density = 2.71 g cc^{-1}). We adopted ρ_{sg} = 2.7 g cc^{-1}. We constrained ϕ_0 and λ using a grid search to minimize the misfit between equation 13 and the index property density at different depths obtained from four IODP boreholes in the Caribbean– sites 998, 999, 1000 and 1001[42] (Supplementary Fig. 1c). We found the best-fitting ϕ_0 is 0.73 and λ is 0.53 (Supplementary Fig. 1d). The best-fitting curve (Supplementary Fig. 1c red curve) is characterized by a faster increase in density with depth than the global average [49] (Supplementary Fig. 1c blue curve), possibly due to the presence of interbedded limestone and volcanic ash in the sediment [42]. We calculated the average sediment density as a function of thickness of the sedimentary layer at each grid point analytically via integration of equation 13 (Supplementary Fig. 1e blue curve).

The crustal thickness at the Caribbean is taken from the differences between basement depth and Moho depth. The Moho depth was obtained with an iterative procedure (see below). A first estimate was generated by assuming the crust to be in local isostatic equilibrium. The density of the regular oceanic crust is assigned as 2.85 g cc^{-1}. The density of the overthickened oceanic crust at the Caribbean is set to 2.85 g cc^{-1}, the same as the global average density of seamounts and oceanic plateaus [50]. Different in composition, the densities of the upper and lower continental crust are assigned as 2.75 and 2.95 g cc^{-1}, respectively. Assuming equal thicknesses of upper and lower continental crust, this gives a similar average continental crustal density of 2.85 g cc^{-1}.

The thickness of the upper mantle is taken from the base of the Moho to 300 km depth. The density of the underlying mantle varies as a function of age, which reflects the thermal contraction of the mantle as it cools down. As a result, older lithospheric ages will produce higher mantle densities. In this study, we used the oceanic crustal age grid from magnetic isochron interpretations of Müller, Sdrolias [51] and the plate cooling model of Richards, Hoggard [52] to obtain the density. As the eruption of the plume head associated with the CLIPs might have rejuvenated the thermal age of the lithosphere, we rescaled the thermal ages at the Caribbean to 100 – 80 Ma based on the ages of the basalts sampled from the oceanic plateau [53] (Fig. 1d). That is, we kept the same pattern of lithospheric age of the Caribbean but shifted it to a younger age range corresponding to the eruption age of the CLIP, instead of the age of formation for the oceanic crust and mantle lithosphere. Our revised ages are consistent with the eastward moving Caribbean plate model of Nerlich, Clark [54]. Since both the age of the CLIP and the age of

formation of the lithosphere are older than 80 Ma, the difference in predicted lithospheric subsidence is relatively minor due to the long-term properties of the plate cooling model [52]. Using the age of formation would give a slightly more positive dynamic uplift of the Caribbean and a slightly stronger dynamic topography gradient but would not materially change our study results. The mantle density under continents was assumed to be constant and equal to 3.3 g cc^{-1} for simplicity.

With this initial setup we used the software package GM-SYS 3D in Oasis Montaj to conduct a structural gravity inversion, adjusting local Moho depths to fit free-air gravity anomalies. The inversion reached convergence after 10 iterations with a gravity misfit less than 0.5 mGal. At a few locations, seismic reflection and refraction studies were able to image the Moho. For example, the Beata ridge, as a prominent bathymetry high within the Caribbean, is well studied in seismic reflection and refraction. Our results of the gravity-constrained Moho suggest the thickest crust (>20km) at the Beata ridge, which are consistent with seismic refraction studies [36, 37, 39]. Comparing the gravity-constrained Moho depth we just obtained to the independently constrained Moho depth from seismic refraction, we found they are highly correlated (Supplementary Fig. 3) with a reduced χ^2 equal to 1.09, from which we can deduce that the uncertainty of the gravity-constrained Moho is no larger than the uncertainty of seismically-constrained Moho. As a final step, we brought the independent seismic constraints [28, 30, 31, 33, 34, 35, 36, 37, 38, 39, 46, 55, 56, 57, 58, 59] into our structural inversion, obtaining a gravity-and-seismic constrained Moho (Fig. 2a). The horizontal resolution of this inverted Moho depends on the resolution of each grids (Supplementary Table 1). In our study, it is ~10 km in the ocean and ~110 km on the continents, which reflects the wider grid space of the global sediment thickness on the continents (Supplementary Table 1).

Supplementary Note 4: Comparison to other published Moho models

Our gravity-and-seismic constrained Moho (Fig. 2a; Source Data) shows the deepest Moho depth (>60 km) at the Andes and the shallowest Moho depth (< 7km) at the Cocos-Nazca spreading center and Cayman trough (Fig. 2a). Within the Caribbean, three prominent features can be clearly identified in our Moho model: the Beata ridge, the Colombia and the Venezuela

basins (Fig. 2a). In the following discussion, we will compare our Moho model with other published models (Supplementary Fig. 2), specifically focusing on these three features.

The models of Crust1.0 [60] and the model of Szwillus, Afonso [61] used the same seismic refraction data but different methodologies. The two models as well as our model agree the eastern Caribbean has shallower Moho. However, our model can more precisely capture the boundary of the regular-thickness oceanic lithosphere imaged from seismic reflection [5] in the Venezuela basin. The overthicknened Beata ridge is also more prominent in our map (BR in Supplementary Fig. 2). The two models show a deeper Moho in the western Caribbean compared to our model. Considering that only few refraction constraints are located on the western Caribbean (Fig. 2a blue boxes), their models might be biased due to interpolation between the shallower Moho in the east Caribbean and the deeper Moho in central America. The GEMMA model [62] also used satellite gravity to invert for Moho depth (Supplementary Fig. 2e) and generally shows a similar trend as our results. The main difference comes from the western Caribbean, where the GEMMA model indicates a similar Moho to the east Caribbean, while our results indicate a deeper Moho. This difference might come from different sediment thickness used in the gravity inversion. Our model includes more recent reflection studies to update the sedimentary thickness of Laske [14] with higher resolution (10 km in our study compared to 110 km in the GEMMA model).

Supplementary Table 1 Map grids used in this study

Layers		Grid	Horizontal Resolution	References
Topography	Continent	Gtop 30	30 arc-sec (~1 km)	DAAC [16]
Bathymetry	Ocean	TerrainBase Global Terrain Model	5 arc-min (~ 8.8 km)	Row and Hastings [17]
Sedimentary Thickness	Continent	Global sediment thickness	1º (~110 km)	Laske [14]
	Ocean	Sediment Thickness of the World's Oceans & Marginal Seas	5 arc-min (~ 8.8 km)	Divins [15]
	Caribbean	Gridded from seismic reflection, refraction and boreholes	5 arc-min (~ 8.8 km)	This Study
Age of the lithosphere	Ocean	Oceanic lithosphere age grid	2 arc-min (~ 3.5 km)	Müller *et al.* [51]
	Caribbean	Müller, Sdrolias [51] rescaled between 100 – 80 Ma based on new age dates from [53]	2 arc-min (~ 3.5 km)	This Study

Supplementary Table 2 Thickness and density of the layers used in the 3D gravity model

Layers	Thickness	Density (g cc^{-1})	Reference of density
Sea water	Sea level to Bathymetry	1.03	Hoggard *et al.* [49]
Sediment	Bathymetry to Basement	1.5-2.7 (Thickness dependent)	This study
Crust	Basement to Moho	2.85	Tetreault and Buiter [50]
Mantle (Continental)	Moho to ~300 km	3.3	Hoggard *et al.* [49]
Mantle (Oceanic)		3.23 – 3.32 (Age dependent)	Richards *et al.* [52]

Supplementary Table 3 Uncertainty propagation analysis of our modeled crustal thickness from crustal density, sediment thickness, and sediment density errors.

	error STD	effect on the Moho Depths	correlation coefficient
crust density	0.096 g cc^{-1}	1081m	r = -1
sediment thickness	40 m	160 m	r = 1
average sediment density	0.0876 g cc^{-1}	75 m	r = -1

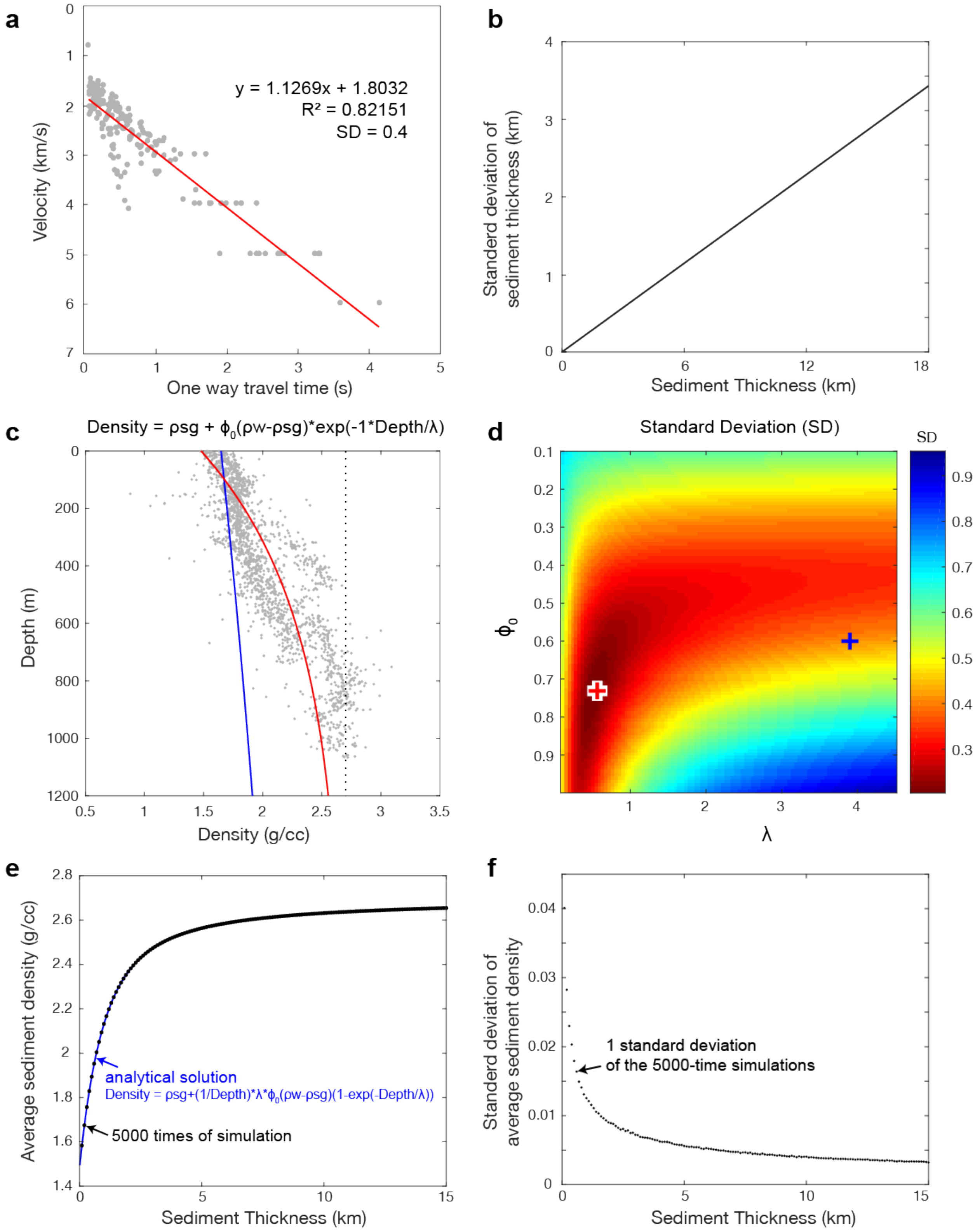

a
y = 1.1269x + 1.8032
R² = 0.82151
SD = 0.4
Velocity (km/s)
One way travel time (s)
b
Standerd deviation of sediment thickness (km)
Sediment Thickness (km)
c
Density = ρsg + ϕ0(ρw-ρsg)*exp(-1*Depth/λ)
Depth (m)
Density (g/cc)
d
Standard Deviation (SD)
SD
ϕ0
λ
e
Average sediment density (g/cc)
analytical solution
Density = ρsg+(1/Depth)*λ*ϕ0(ρw-ρsg)(1-exp(-Depth/λ))
5000 times of simulation
Sediment Thickness (km)
f
Standard deviation of average sediment density
1 standard deviation of the 5000-time simulations
Sediment Thickness (km)

Supplementary Fig. 1 Uncertainty estimations. (a) Velocity structure at the Caribbean summarized from where seismic refraction and reflection are both conducted [31, 32, 33, 34, 46]. The uncertainty of the velocity structure contributes the uncertainty of the sediment thickness in (b), assuming little uncertainty of seismic travel time measurements. (c) Sediment depth-density relations regressed from the index property density of IODP boreholes [42] in the Caribbean. The blue curve shows the global best-fit curve from Hoggard, White [49]. The red curve shows the best fit regression with two parameters ϕ_0 (initial porosity) and λ (the compaction decay length) obtained from grid search in (d). The red and blue crosses correspond to the value of the parameters used in the two curves in (c). (e) The average sediment density as a function of sediment thickness. The blue curve is the analytical solution of integrating equation 13. We also simulated average density by randomly selecting the sediment density every 10 m (see Methods for details). The average of the 5,000-time simulation is shown as black dots. The standard deviation of those simulations is assigned to be the uncertainty of average sediment density in (f).

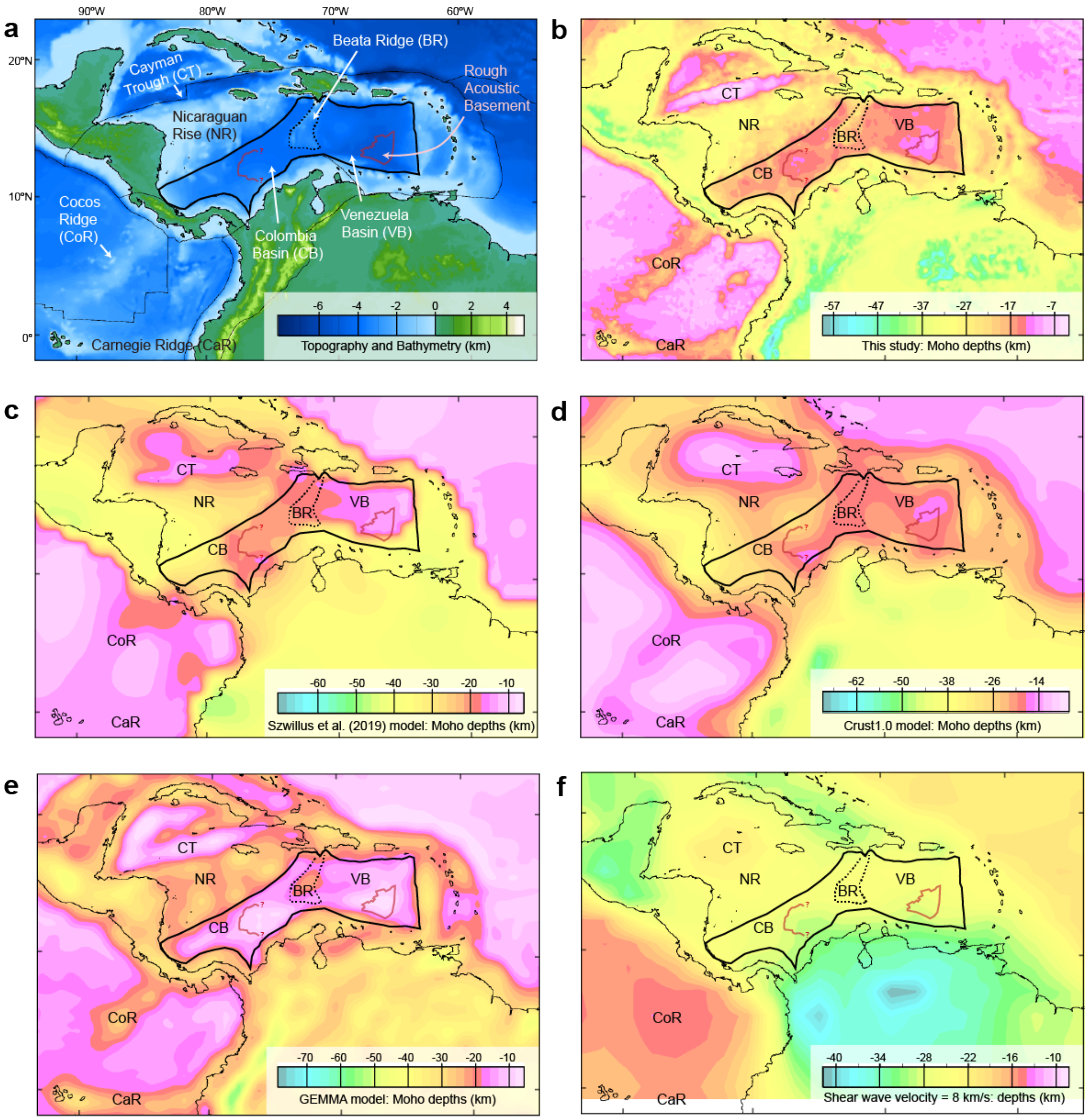

Supplementary Fig. 2 Comparison of our Caribbean Moho depth model to previously published models. (a) Caribbean topography and bathymetry for reference. CT: Cayman Trough. NR: Nicaraguan Rise. CoR: Cocos Ridge. CaR: Carnegie Ridge. The thick crust of the Beata Ridge (BR) is bounded by black dash line. The thin crust of the Colombia basin (CB) and the Venezuela basin (VB) are shown by red lines. The thick black line is for reference. (b) Our gravity-and-seismic constrained Moho. (c) The Moho model of Szwillus, Afonso [61] and (d) the

Crust1.0 model [60], both used seismic refraction data but with different interpolation approaches. (e) The GEMMA model [62] based on satellite gravity inversion. (f) Moho defined from a 8 km s^{-1} seismic velocity isosurface from the surface wave tomography of Gaite, Villaseñor [63].

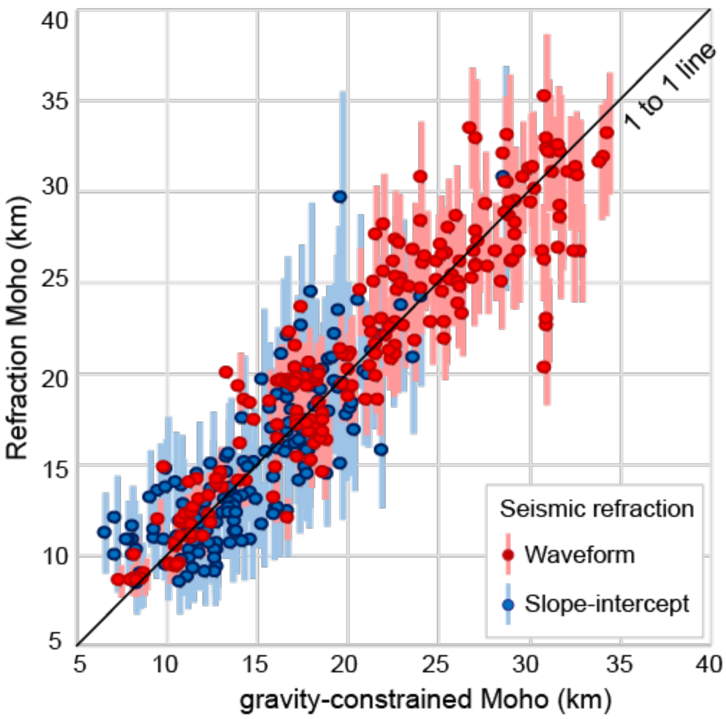

Supplementary Fig. 3 Comparison of Moho depths derived from seismic refraction and from a preliminary model obtained by using only gravity constraints. The locations of refraction stations are shown in Fig. 2a. The uncertainty of the refraction Moho depth is assigned to be ~10% of the crustal thickness for waveform-modelled Moho [61] (i.e. seismic wide-angle experiments) and ~20% for slope-intercept Moho [64] (i.e. vintage seismic reflection experiments) (see Supplementary Note 3 for details). Our comparison suggests that even before seismic constraints were applied, our gravity-constrained Moho depths were highly consistent with the refraction-derived Moho (reduced $\chi 2$ equal to 1.09). We thus assign an uncertainty to our gravity-and-seismic Moho depth to be ~10% of the crustal thickness.

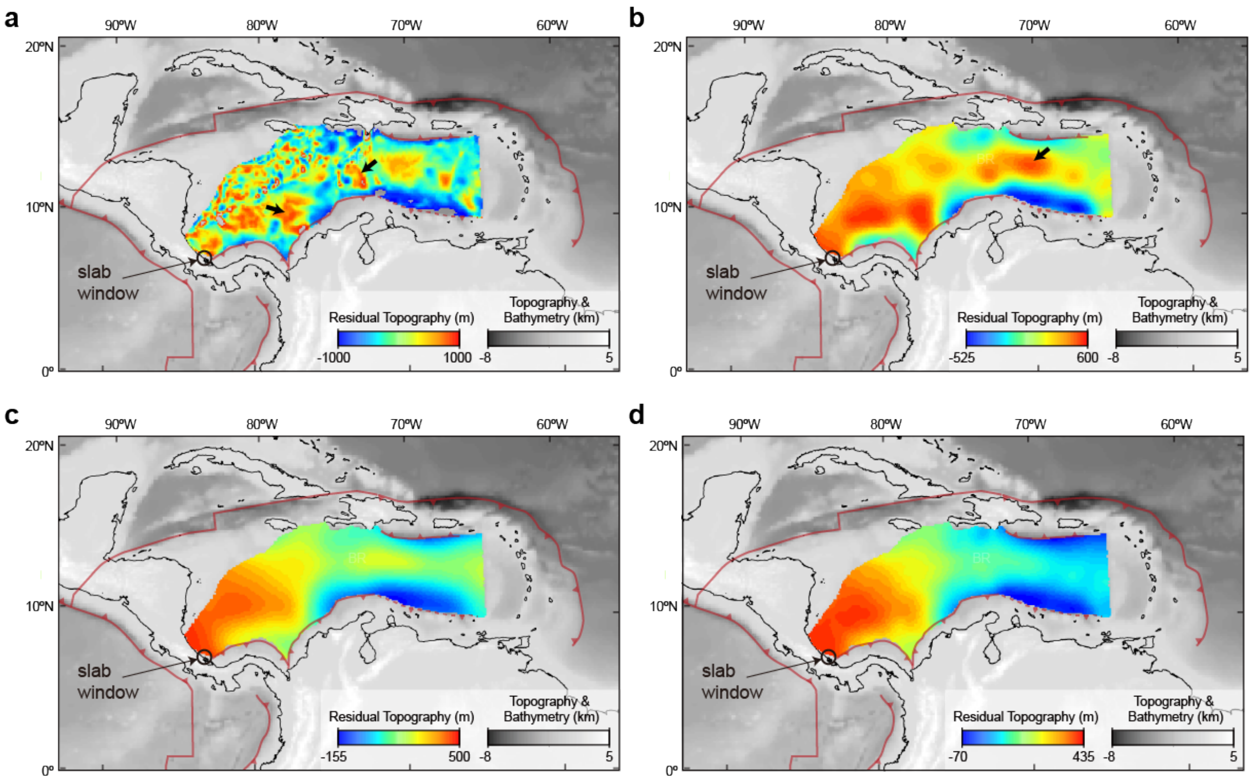

Supplementary Fig. 4. Effect of low-pass filtering on the residual basement depth. (a) Unfiltered residual basement depth. It is worth noting that, while most of the short-scale features are probably due to flexural effects, some mid-scale topographic highs (black arrows) align with slow seismic anomalies in the asthenosphere (Fig. 2d), suggesting that they might be dynamically supported. (b) A 4th-order Butterworth low-pass filter with 300 km corner wavelength was applied to the residual basement depth in (a). Part of the signal can still be attributed to lithospheric flexure. For example, the topographic high in the Venezuela basin (black arrow) might be the forebulge of the double-verging subduction zones to its north and south [5] (Fig. 1a). Indeed, a recent analysis of the power spectrum of Earth's free-air gravity anomalies shows a dominant role of flexural isostasy for wavelengths ≤ 300 km [65]. (c) A 2nd - order Butterworth low-pass filter with 600 km corner wavelength was applied to the residual basement depth in (a). This filter is comparable to the one used by Nerlich, Clark [66]. (d) A 2nd -

order Butterworth low-pass filter with 1000 km corner wavelength was applied to the residual basement depth in (a), and the dynamic signal is prominent.

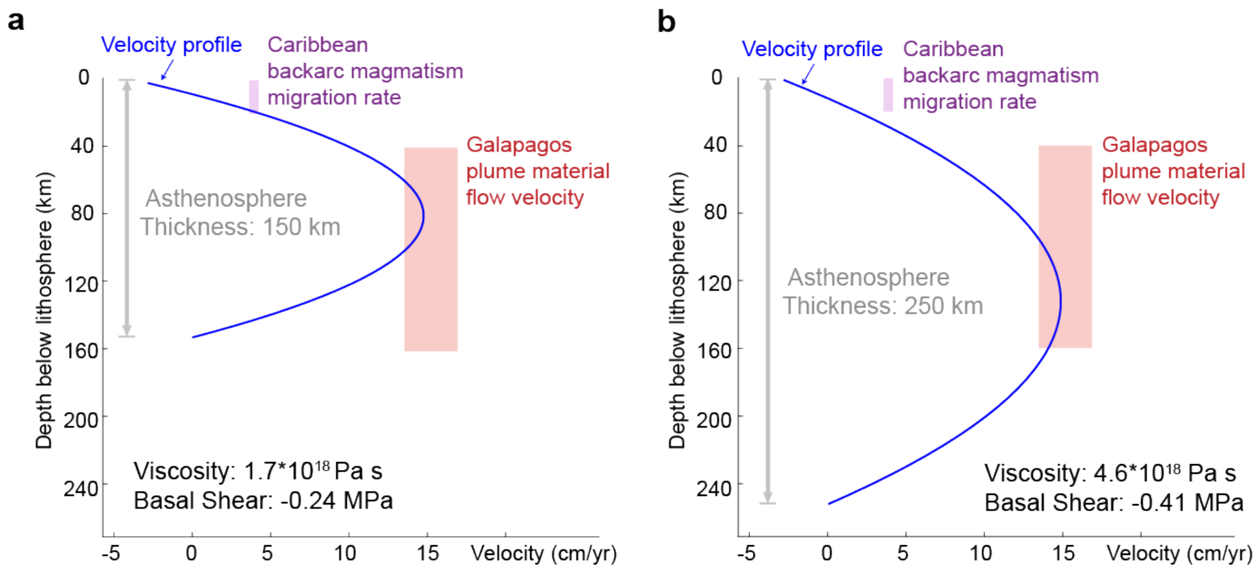

Supplementary Fig. 5 Sensitivity tests showing the effect of a different asthenospheric thickness on the best-fit velocity profile and its associated asthenospheric viscosity. Our preferred viscosity of $3*10^{18}$ Pa·s with 200 km thick asthenosphere would change by 50% if the asthenosphere thickness imaged in seismic tomographic varied by 25% from 200 km to (a) 150 km or to (b) 250 km, which is somewhat possible given the limited resolution of seismic tomography. The two boxes are independent constraints of flow velocity (same as Fig. 4).

Supplementary References

1. Allen RW*, et al.* The role of arc migration in the development of the Lesser Antilles: A new tectonic model for the Cenozoic evolution of the eastern Caribbean. *Geology* **47**, 891-895 (2019).

2. Boschman LM, van der Wiel E, Flores KE, Langereis CG, van Hinsbergen DJJ. The Caribbean and Farallon Plates Connected: Constraints From Stratigraphy and Paleomagnetism of the Nicoya Peninsula, Costa Rica. *Journal of Geophysical Research: Solid Earth* **124**, 6243-6266 (2019).

3. Pindell JL, Kennan L. Tectonic evolution of the Gulf of Mexico, Caribbean and northern South America in the mantle reference frame: an update. *Geological Society, London, Special Publications* **328**, 1-55 (2009).

4. Cardona A*, et al.* Early-subduction-related orogeny in the northern Andes: Turonian to Eocene magmatic and provenance record in the Santa Marta Massif and Rancheria Basin, northern Colombia. *Terra Nova* **23**, 26-34 (2011).

5. Kroehler ME, Mann P, Escalona A, Christeson GL. Late Cretaceous-Miocene diachronous onset of back thrusting along the South Caribbean deformed belt and its importance for understanding processes of arc collision and crustal growth. *Tectonics* **30**, TC6003 (2011).

6. Luzieux LDA, Heller F, Spikings R, Vallejo CF, Winkler W. Origin and Cretaceous tectonic history of the coastal Ecuadorian forearc between 1°N and 3°S: Paleomagnetic, radiometric and fossil evidence. *Earth and Planetary Science Letters* **249**, 400-414 (2006).

7. Rogers RD, Mann P, Emmet PA, Venable ME, Mann P. Colon fold belt of Honduras: Evidence for Late Cretaceous collision between the continental Chortis block and intra-oceanic Caribbean arc. In: *Geologic and Tectonic Development of the Caribbean Plate Boundary in Northern Central America*. (Geological Society of America, Boulder, 2007).

8. Wegner W, Wörner G, Harmon RS, Jicha BR. Magmatic history and evolution of the Central American Land Bridge in Panama since Cretaceous times. *GSA Bulletin* **123**, 703-724 (2011).

9. Abratis M, Wörner G. Ridge collision, slab-window formation, and the flux of Pacific asthenosphere into the Caribbean realm. *Geology* **29**, 127-130 (2001).

10. Lonsdale P. Creation of the Cocos and Nazca plates by fission of the Farallon plate. *Tectonophysics* **404**, 237-264 (2005).

11. Rooney TO, Morell KD, Hidalgo P, Fraceschi P. Magmatic consequences of the transition from orthogonal to oblique subduction in Panama. *Geochemistry, Geophysics, Geosystems* **16**, 4178-4208 (2015).

12. Johnston ST, Thorkelson DJ. Cocos-Nazca slab window beneath Central America. *Earth and Planetary Science Letters* **146**, 465-474 (1997).

13. Gazel E*, et al.* Plume–subduction interaction in southern Central America: Mantle upwelling and slab melting. *Lithos* **121**, 117-134 (2011).

14. Laske G. A global digital map of sediment thickness. *Eos Trans AGU* **78**, F483 (1997).

15. Divins D. Total sediment thickness of the world's oceans & marginal seas. (NOAA National Geophysical Data Center.) https://www.ngdc.noaa.gov/mgg/sedthick/sedthick.html (2003).

16. DAAC L. Global 30 Arc-Second Elevation Data Set GTOPO30. (Land Process Distributed Active Archive Center). DOI: /10.5066/F7DF6PQS (2004).

17. Row L, Hastings D. TerrainBase: Worldwide digital terrain data (NOAA National Geophysical Data Center) (1995).

18. Bowland CL. Depositional history of the western Colombian Basin, Caribbean Sea, revealed by seismic stratigraphy. *GSA Bulletin* **105**, 1321-1345 (1993).

19. Duncan DS, Hine AC, Droxler AW. Tectonic controls on carbonate sequence formation in an active strike–slip setting: Serranilla Basin, Northern Nicaragua Rise, Western Caribbean Sea. *Marine Geology* **160**, 355-382 (1999).

20. Sanchez J, Mann P, Carvajal-Arenas LC, Bernal-Olaya R. Regional transect across the western Caribbean Sea based on integration of geologic, seismic reflection, gravity, and magnetic data. *AAPG Bulletin* **103**, 303-343 (2019).

21. Barboza G, Fernandez JA, Barrientos J, Bottazzi G. Costa Rica; petroleum geology of the Caribbean margin. *The Leading Edge* **16**, 1787-1794 (1997).

22. Bernal-Olaya R, Sanchez J, Mann P, Murphy M, Bartolini C, Mann P. Along-strike Crustal Thickness Variations of the Subducting Caribbean Plate Produces Two Distinctive Styles of Thrusting in the Offshore South Caribbean Deformed Belt, Colombia. In: *Petroleum Geology and Potential of the Colombian Caribbean Margin*. (American Association of Petroleum Geologists, Tulsa, 2015).

23. Granja Bruña JL*, et al.* Morphostructure at the junction between the Beata ridge and the Greater Antilles island arc (offshore Hispaniola southern slope). *Tectonophysics* **618**, 138-163 (2014).

24. Londono J, Schiek C, Biegert E, Bartolini C, Mann P. Basement Architecture of the Southern Caribbean Basin, Guajira Offshore, Colombia. In: *Petroleum Geology and Potential of the Colombian Caribbean Margin*. (American Association of Petroleum Geologists, Tulsa, 2015).

25. Ott BM. *Cretaceous-Cenozoic tectonostratigraphic evolution of the Nicaraguan Rise based on seismic reflection, wells, and potential fields data*. (University of Houston, Houston, 2015).

26. Carvajal-Arenas LC. *Hydrocarbon Prospectivity of the Nicaraguan Rise and Colombian Basin, Western Caribbean Sea*. (University of Houston, Houston, 2017).

27. Basabe Triana YD. Sedimentary Megasequences of Colombian Basin, Offshore Colombia.). The University of Arizona (2018).

28. Kopp H*, et al.* Deep structure of the central Lesser Antilles Island Arc: Relevance for the formation of continental crust. *Earth and Planetary Science Letters* **304**, 121-134 (2011).

29. Clark SA, Zelt CA, Magnani MB, Levander A. Characterizing the Caribbean–South American plate boundary at 64°W using wide-angle seismic data. *Journal of Geophysical Research: Solid Earth* **113**, B07401 (2008).

30. Bezada MJ, Magnani MB, Zelt CA, Schmitz M, Levander A. The Caribbean–South American plate boundary at 65°W: Results from wide-angle seismic data. *Journal of Geophysical Research: Solid Earth* **115**, B08402 (2010).

31. Houtz RE, Ludwig WJ. Structure of Colombia Basin, Caribbean Sea, from profiler-sonobuoy measurements. *Journal of Geophysical Research* **82**, 4861-4867 (1977).

32. Ludwig WJ, Houtz RE, Ewing JI. Profiler-sonobuoy measurements in Colombia and Venezuela basins, Caribbean Sea. *AAPG Bulletin* **59**, 115-123 (1975).

33. Christeson GL, Mann P, Escalona A, Aitken TJ. Crustal structure of the Caribbean–northeastern South America arc-continent collision zone. *Journal of Geophysical Research: Solid Earth* **113**, B08104 (2008).

34. Magnani MB, Zelt CA, Levander A, Schmitz M. Crustal structure of the South American–Caribbean plate boundary at 67°W from controlled source seismic data. *Journal of Geophysical Research: Solid Earth* **114**, B02312 (2009).

35. Officer CB, Ewing JI, Hennion JF, Harkrider DG, Miller DE. Geophysical investigations in the eastern Caribbean: Summary of 1955 and 1956 cruises. *Physics and Chemistry of the Earth* **3**, 17-109 (1959).

36. Ewing J, Antoine J, Ewing M. Geophysical measurements in the western Caribbean Sea and in the Gulf of Mexico. *Journal of Geophysical Research* **65**, 4087-4126 (1960).

37. Edgar NT, Ewing JI, Hennion J. Seismic refraction and reflection in Caribbean Sea. *AAPG Bulletin* **55**, 833-870 (1971).

38. Diebold JB, Stoffa PL, Buhl P, Truchan M. Venezuela Basin crustal structure. *Journal of Geophysical Research: Solid Earth* **86**, 7901-7923 (1981).

39. Núñez D, Córdoba D, Cotilla MO, Pazos A. Modeling the Crust and Upper Mantle in Northern Beata Ridge (CARIBE NORTE Project). *Pure and Applied Geophysics* **173**, 1639-1661 (2016).

40. Lewis J, Mattietti GK, Perfit M, Kamenov G. Geochemistry and petrology of three granitoid rock cores from the Nicaraguan Rise, Caribbean Sea: implications for its composition, structure and tectonic evolution. *Geologica Acta* **9**, 467-479 (2011).

41. Macellari CE, Tankard AJ, Soruco RS, Welsink HJ. Cenozoic Sedimentation and Tectonics of the Southwestern Caribbean Pull-Apart Basin, Venezuela and Colombia. In: *Petroleum Basins of South Americ*. (American Association of Petroleum Geologists, Tulsa, 1995).

42. Sigurdsson H*, et al. Proceedings of the Ocean Drilling Program; Initial reports; Caribbean ocean history and the Cretaceous/Tertiary boundary event; covering Leg 165 of the cruises of the Drilling Vessel JOIDES Resolution, Miami, Florida, to San Juan, Puerto Rico, sites 998-1002, 19 December 1995-17 February 1996*. (Texas A & M University, College Station, 1997).

43. Edgar NT*, et al. Initial reports of the Deep Sea Drilling Project, covering Leg 15 of the cruises of the drilling vessel Glomar Challenger, San Juan, Puerto Rico to Cristobal, Panama; December 1970-February 1971*. (Texas A & M University, College Station, 1973).

44. Ysaccis B. R. *Tertiary evolution of the northeastern Venezuela offshore*. (Rice University, Huston, 1998).

45. Villarreal MG. *Seismic studies of the western Caribbean : wide-angle seismic refraction analysis of the Nicaragua Rise, and seismic reflection imaging of the Hess Escarpment*. (University of Wyoming, Laramie, 2012).

46. Clark SA, Levander A, Magnani MB, Zelt CA. Negligible convergence and lithospheric tearing along the Caribbean–South American plate boundary at 64°W. *Tectonics* **27**, TC6013 (2008).

47. Sandwell DT, Müller RD, Smith WHF, Garcia E, Francis R. New global marine gravity model from CryoSat-2 and Jason-1 reveals buried tectonic structure. *Science* **346**, 65-67 (2014).

48. Amante C, Eakins BW. ETOPO1 Global Relief Model converted to PanMap layer format. (NOAA-National Geophysical Data Center, PANGAEA) https://doi.org/10.1594/PANGAEA.769615 (2009).

49. Hoggard MJ, White N, Al-Attar D. Global dynamic topography observations reveal limited influence of large-scale mantle flow. *Nature Geoscience* **9**, 456-463 (2016).

50. Tetreault JL, Buiter SJH. Future accreted terranes: a compilation of island arcs, oceanic plateaus, submarine ridges, seamounts, and continental fragments. *Solid Earth* **5**, 1243-1275 (2014).

51. Müller RD, Sdrolias M, Gaina C, Roest WR. Age, spreading rates, and spreading asymmetry of the world's ocean crust. *Geochemistry, Geophysics, Geosystems* **9**, Q04006 (2008).

52. Richards FD, Hoggard MJ, Cowton LR, White NJ. Reassessing the Thermal Structure of Oceanic Lithosphere With Revised Global Inventories of Basement Depths and Heat Flow Measurements. *Journal of Geophysical Research: Solid Earth* **123**, 9136-9161 (2018).

53. Whattam SA, Stern RJ. Late Cretaceous plume-induced subduction initiation along the southern margin of the Caribbean and NW South America: The first documented example with implications for the onset of plate tectonics. *Gondwana Research* **27**, 38-63 (2015).

54. Nerlich R, Clark SR, Bunge H-P. Reconstructing the link between the Galapagos hotspot and the Caribbean Plateau. *GeoResJ* **1-2**, 1-7 (2014).

55. Gazel E*, et al.* Continental crust generated in oceanic arcs. *Nature Geoscience* **8**, 321-327 (2015).

56. Sallarès V, Charvis P, Flueh ER, Bialas J. Seismic structure of Cocos and Malpelo Volcanic Ridges and implications for hot spot-ridge interaction. *Journal of Geophysical Research: Solid Earth* **108**, 2564 (2003).

57. Van Avendonk HJA, Harding AJ, Orcutt JA, McClain JS. Contrast in crustal structure across the Clipperton transform fault from travel time tomography. *Journal of Geophysical Research: Solid Earth* **106**, 10961-10981 (2001).

58. Ewing JI, Officer CB, Johnson HR, Edwards RS. Geophysical investigations in the eastern Caribbean: Trinidad shelf, Tobago trough, Barbados ridge, Atlantic Ocean. *GSA Bulletin* **68**, 897-912 (1957).

59. Northrop J, Ransone M. Some Seismic Profiles near the Western End of the Puerto Rico Trench. *J Gen Physiol* **45**, 243-251 (1962).

60. Laske G, Masters G, Ma Z, Pasyanos M. Update on CRUST1. 0—A 1-degree global model of Earth's crust. *Geophysical Research Abstracts* **15**, Abstract EGU2013–2658 (2013).

61. Szwillus W, Afonso JC, Ebbing J, Mooney WD. Global Crustal Thickness and Velocity Structure From Geostatistical Analysis of Seismic Data. *Journal of Geophysical Research: Solid Earth* **124**, 1626-1652 (2019).

62. Reguzzoni M, Sampietro D. GEMMA: An Earth crustal model based on GOCE satellite data. *International Journal of Applied Earth Observation and Geoinformation* **35**, 31-43 (2015).

63. Gaite B, Villaseñor A, Iglesias A, Herraiz M, Jiménez-Munt I. A 3-D shear velocity model of the southern North American and Caribbean plates from ambient noise and earthquake tomography. *Solid Earth* **6**, 271-284 (2015).

64. White RS, McKenzie D, O'Nions RK. Oceanic crustal thickness from seismic measurements and rare earth element inversions. *Journal of Geophysical Research: Solid Earth* **97**, 19683-19715 (1992).

65. Watts AB, Moore JDP. Flexural Isostasy: Constraints From Gravity and Topography Power Spectra. *Journal of Geophysical Research: Solid Earth* **122**, 8417-8430 (2017).

66. Nerlich R, Clark SR, Bunge H-P. An outlet for Pacific mantle: The Caribbean Sea? *GeoResJ* **7**, 59-65 (2015).
